# Reduced C9orf72 protein levels in frontal cortex of amyotrophic lateral sclerosis and frontotemporal degeneration brain with the *C9ORF72* hexanucleotide repeat expansion^☆^

**DOI:** 10.1016/j.neurobiolaging.2014.01.016

**Published:** 2014-07

**Authors:** Adrian J. Waite, Dirk Bäumer, Simon East, James Neal, Huw R. Morris, Olaf Ansorge, Derek J. Blake

**Affiliations:** a Institute of Psychological Medicine and Clinical Neurosciences, MRC Centre for Neuropsychiatric Genetics and Genomics, School of Medicine, Cardiff University, Cathays, Cardiff, UK; b Nuffield Department of Clinical Neurosciences, University of Oxford, John Radcliffe Hospital, Oxford, UK; c Department of Pathology, School of Medicine, Cardiff University, Cardiff, UK; d Department of Clinical Neuroscience, UCL Institute of Neurology, Royal Free Hospital, London, UK

**Keywords:** Amyotrophic lateral sclerosis, C9orf72, Frontotemporal dementia, Frontotemporal lobar degeneration, Repeat expansion, Southern blotting

## Abstract

An intronic G_4_C_2_ hexanucleotide repeat expansion in *C9ORF72* is a major cause of amyotrophic lateral sclerosis and frontotemporal lobar degeneration. Several mechanisms including RNA toxicity, repeat-associated non-AUG translation mediated dipeptide protein aggregates, and haploinsufficiency of C9orf72 have been implicated in the molecular pathogenesis of this disorder. The aims of this study were to compare the use of two different Southern blot probes for detection of repeat expansions in an amyotrophic lateral sclerosis and frontotemporal lobar degeneration pathological cohort and to determine the levels of C9orf72 transcript variants and protein isoforms in patients versus control subjects. Our Southern blot studies identified smaller repeat expansions (250–1800 bp) that were only detectable with the flanking probe highlighting the potential for divergent results using different Southern blotting protocols that could complicate genotype–phenotype correlation studies. Further, we characterize a new C9orf72 antibody and show for the first time decreased C9orf72 protein levels in the frontal cortex from patients with a pathological hexanucleotide repeat expansion. These data suggest that a reduction in C9orf72 protein may be a consequence of the disease.

## 1 Introduction

An intronic G_4_C_2_ hexanucleotide repeat expansion in the first intron or alternative promoter of the uncharacterized *C9ORF72* gene was identified as the cause of amyotrophic lateral sclerosis (ALS), frontotemporal lobar degeneration (FTLD), and ALS-FTLD syndrome linked to chromosome 9p21 (DeJesus-Hernandez et al., 2011; Gijselinck et al., 2012; Renton et al., 2011). Repeat-primed polymerase chain reaction detection of hexanucleotide expansions in a large cohort of ALS and FTLD cases identified expansions in 8% of sporadic ALS, 39% of familial ALS, 7% sporadic of FTLD, and 25% of familial FTLD cases (Majounie et al., 2012b). Expansions have also been reported in confirmed cases of Alzheimer's disease (Majounie et al., 2012a), corticobasal, and ataxic syndromes (Lindquist et al., 2013; Snowden et al., 2012). Although commonly used polymerase chain reaction (PCR)-based methods allow the detection of repeat expansions, they are unable to size the expansions hampering further genotype–phenotype studies. Southern blot protocols allow the sizing of the expansions, however only a limited number have been published to date (Beck et al., 2013; Buchman et al., 2013; DeJesus-Hernandez et al., 2011; van Blitterswijk et al., 2013). Studies analyzing sufficient samples numbers have reported a correlation of modal repeat size with age at clinical onset of symptoms (Beck et al., 2013; van Blitterswijk et al., 2013), however further cross-sectional studies are required to strengthen these findings.

Preliminary studies suggest a complex pathological mechanism associated with the *C9ORF72* hexanucleotide repeat expansion (Ash et al., 2013; DeJesus-Hernandez et al., 2011; Mori et al., 2013b). A number of reports have shown decreased levels of C9orf72 transcripts in expansion cases suggesting loss-of-function as one such mechanism (Ciura et al., 2013; Gijselinck et al., 2012; Mori et al., 2013b). However, the effect on protein isoform levels has yet to be determined.

In this study we compared the use of repeat-derived and single-copy repeat flanking Southern blot probes for detection of hexanucleotide expansions in an ALS-FTD pathological cohort. Furthermore, we generated and characterized a custom anti-C9orf72 polyclonal antibody to investigate C9orf72 protein abundance in hexanucleotide repeat expansion carriers.

## 2 Methods

### 2.1 Subjects

Subjects were recruited as part of a study of familial ALS and FTLD in Wales (REC for Wales 09/MRE09/35). Additional cases were identified in the Oxford Brain Bank. (REC for England 07/H0606/85). All cases showed ALS or FTLD neuropathology characteristic of *C9ORF72* mutation carriers, namely a combination of TDP-43 positive and non-TDP-43 p62-positive inclusions. Following consent, blood samples were obtained for DNA extraction and the establishment of lymphoblastoid cell lines. Tissue was obtained at postmortem for neuropathological assessment and molecular pathological study. Patients had a primary diagnosis of ALS (n = 6), pure FTLD (n = 1), and ALS-FTLD (n = 9). A family history of ALS and/or FTLD was present in 11 cases. Three members came from the Gwent kindred in whom the *C9ORF72* expansion was previously identified (Renton et al., 2011) (clinical information in Supplementary Table 1).

### 2.2 Southern blot

The *C9ORF72* single-copy repeat flanking probe sequence (Southern blot restriction digests and probe location shown in Supplementary Fig. 1) was PCR amplified (primers in Supplementary Table 4) from control genomic DNA (gDNA) and cloned into pGEM-T (Promega, Southampton, UK). Digoxigenin (DIG)-labeled probe was amplified from purified plasmid using the PCR DIG Probe Synthesis Kit (Roche Applied Science, Burgess Hill, UK). The PCR product was purified using a gel extraction kit (Qiagen, Manchester, UK) before use.

gDNA (5–7.5 μg) was digested overnight with EcoRI-HF (40 U)/BamHI-HF (40 U) or only EcoRI (40 U). Next day, digests were supplemented with 20 U additional enzyme(s) and incubated for a further 2 hours. Southern blotting reagents were purchased from Roche Applied Science unless otherwise stated. Digested DNA was separated by electrophoresis on 0.8% agarose gels and transferred onto positively charged nylon membranes by capillary blotting (0.4 M NaOH) followed by baking at 80 °C for 2 hours. Membranes were pre-hybridized with DIG Easy Hyb buffer (3 hours at 46 °C) before overnight hybridization at 46 °C with hybridization buffer containing 1 μL/mL *C9ORF72* flanking DIG-probe. Membranes were washed in 2× saline sodium citrate with 0.1% (wt/vol) sodium dodecyl sulfate (SDS) while the oven was ramped from 46 °C to 65 °C. Two 15 minutes high stringency washes were performed in 0.5 × saline sodium citrate + 0.1% (wt/vol) SDS at 65 °C. Detection of hybridized DIG-probe was carried out using the DIG wash and block kit and CDP-Star reagent as a chemiluminescent substrate. Signals were visualized on Kodak X-OMAT LS film after 1–4 hours. Estimation of repeat expansion ranges were performed as described previously (Beck et al., 2013). Southern blots using a DIG-labeled 5xGGGGCC probe (hexanucleotide pentamer probe) were performed as previously described with stringency washes and probe detection as described previously (Beck et al., 2013).

### 2.3 Reverse transcriptase-PCR and quantitative PCR

Two independent quantitative PCR (qPCR) experiments were carried out. For the first set, total RNA was extracted from tissue samples using TRI Reagent (Ambion, Paisley, UK) and treated with DNA-free DNase (Ambion) before quantification and quality assessment. RNA integrity (RIN) was checked on an Agilent 2100 Bioanalyzer with a RIN cutoff of 4 for use in qPCR. Cases and control subjects had comparable RIN ranges. RNA was reverse transcribed to complementary DNA (cDNA) using random decamers and the Retroscript cDNA synthesis kit (Ambion). qPCR was performed using Brilliant III ultra-fast SYBR Green PCR reagent (Agilent Technologies, Wokingham, UK) following manufacturer's instructions and custom *C9ORF72* primers (Supplementary Table 3) and analyzed on an ABI 7900HT system (Applied Biosystems, Paisley, UK). Primer efficiencies were determined for primer pairs by generation of a standard curve before use on experimental samples. Three technical replicates were performed for each sample. Relative quantification was performed using the ΔΔC_t_ method after normalization to *ACTNB*. Statistical significance of ΔC_t_ values was determined using a 1-tailed *t* test assuming unequal variance.

For the second set of experiments, RNA was extracted from frozen brain tissue using the Qiagen RNeasy Lipid Tissue Mini Kit with on-column DNAse treatment to remove genomic DNA. RNA quality was then assessed as previously mentioned (RIN values 4.1–7.3 with no significant difference between groups). cDNA was generated from 3.5 μg RNA input using a SuperScript VILO cDNA synthesis kit with random hexamer primers (Life Technologies, Paisley, UK). RT-PCR was carried out with forward primers in *C9ORF72* exon 1a (GTCAAACAGCGACAAGTTCCG) and 1b (AGGCGCAGGCGGTGGCGAGTG) and reverse primer in exon 2 (ACCTGTTCTGTCTTTGGAGCC) using Sigma Taq polymerase in a standard PCR reaction, annealing temperature 59 °C, 30 cycles. cDNA input for the exon 1 b PCR was 50% of that for the exon 1a PCR. PCR products were resolved on a 1.5% agarose gel stained using GelRed. qPCR was carried out using a previously described custom made TaqMan assay detecting the exon 1B containing variant 1 (NM_018325.3) (DeJesus-Hernandez et al., 2011) (Applied Biosystems). Reactions were carried out in triplicate on a Rotor-Gene Q cycler (Qiagen) using TaqMan Gene Expression Mastermix (Applied Biosystems). Data were analyzed using the ΔΔC_t_ method with GAPDH (assay ID Hs00266705, Applied Biosystems) as a reference gene. Ct values for GAPDH were evenly distributed across genotypes and regions examined. ΔC_t_ values were compared using an unpaired *t* test not assuming equal variances.

### 2.4 Antibody production

The custom anti-C9orf72 rabbit polyclonal antibody (C9-3721) was generated from an N-terminal thioredoxin fusion of C9orf72 short isoform (Thx-C9orf72-Short). Human C9orf72 short was amplified from SH-SY5Y cDNA (primers in Supplementary Table 4). The PCR product was digested and cloned into the EcoRI/NotI sites of pET32b (Clontech, Mountain View, CA, USA). Construct was sequence verified before use. Thx-C9orf72-Short expression was induced in transformed BL21(DE3) *Escherichia coli* (Agilent Technologies) using 1 mM isopropyl β-D-1-thiogalactopyranoside (IPTG) for 3 hours at 37 °C. Thx-C9orf72-Short was purified using HisPur resin according to the manufacturer's instructions (Thermo Fisher Scientific, Loughborough, UK) following solubilization in sonication buffer (20 mM Tris pH 8.0, 100 mM NaCl, 0.1% Triton X-100) using a Vibra-Cell Ultrasonic Processor (Sonics & Materials Inc, Newtown, CT, USA). Purified fusion protein was used as the antigen for custom rabbit polyclonal antibody generation (Covalab S.A.S, Cambridge, UK). Custom antisera were immunoaffinity purified against the antigen following pre-absorption against thioredoxin and glial fibrillary acidic protein (Thx-GFAP expressed in BL21(DE3) transformed with GFAP (pET32a)).

### 2.5 Western blotting

Protein extracts were separated by SDS-PAGE and immunoblotted as previously described (Esapa et al., 2007). Primary antibodies were incubated overnight at 4 °C. C9orf72 commercial antibodies were used at 1:100 dilutions, custom anti-C9orf72 C9-3721 at 1:250, anti-c-myc 9E10 at 1:350 and anti-β-actin (Sigma-Aldrich, Dorset, UK) at 1:2500. After washing the membranes were incubated with IRDye 800 anti-mouse IgG (Rockland Immunochemicals, Gilbertsville, PA, USA) and Alexa Fluor 680 anti-rabbit IgG (Life Technologies) secondary antibodies. Simultaneous 2-color detection was performed using an Odyssey infrared imaging system (LI-COR Biosciences UK Ltd, Cambridge, UK). For quantification samples were run in triplicate with the inclusion of a recombinant C9orf72 protein blot standardizer. Membranes were incubated with C9-3721 and anti-β-actin antibodies before incubation with appropriate IRDye-conjugated secondary antibodies. Specific quantification of the approximate 48 kDa full length C9orf72 isoform (indicated by arrowheads on the immunoblot images) was performed using the details view in Odyssey application software with the average background method and normalizing to endogenous β-actin signal. Blots were normalized using the full-length recombinant C9orf72 standardizer (annotated as myc-C9 LF in the figure panels) before collation of the normalized signal intensity data. Statistical significance of normalized intensity values was determined using a 1-tailed *t* test assuming unequal variance.

## 3 Results

### 3.1 Comparative Southern blot detection of *C9ORF72* hexanucleotide repeat expansions

Since the discovery of the *C9ORF72* hexanucleotide repeat expansion, most studies have used PCR based methods for the detection of repeat expansions (DeJesus-Hernandez et al., 2011). Initial genetic analysis of the pathological material also used these methods (Supplementary Table 1) with all expansion positive cases showing a single detectable allele by flanking PCR and extended sawtooth repeat-primed PCR product of >30 peaks. A limited number of studies have used Southern blotting for analysis of repeat expansion size using both repeat-derived and single-copy repeat flanking probes (Beck et al., 2013; Buchman et al., 2013; DeJesus-Hernandez et al., 2011; van Blitterswijk et al., 2013). For the purpose of estimating repeat size in our pathological cohort we compared the use of a novel repeat flanking Southern probe (single copy probe) and a published hexanucleotide probe (hexanucleotide pentamer probe).

The hexanucleotide probe showed increased sensitivity of detection for large repeat expansions compared with the single-copy probe, with overlap in signal representing modal repeat sizes (Fig. 1). Cerebellar samples (Fig. 1B) consistently showed reduced modal expansion sizes when compared with the frontal cortex samples (Fig. 1A). Southern blot analysis of multiple brain and peripheral tissue samples from a single affected individual (ALS15) demonstrated a reduced modal expansion size range in cerebellar tissue compared with other brain regions examined (Supplementary Fig. 2). Peripheral tissues such as spleen, skeletal muscle, and blood showed an extended repeat expansion size range between that observed in frontal cortex and cerebellum.

Southern blot analysis of cerebellar tissue using both probes (Fig. 1B and C) resulted in a similar pattern of expansion sizes across most of the pathological samples. Regression analysis of the approximate median repeat sizes from both Southern methods showed evidence of linear correlation (R^2^ = 0.89, F significance = 1.5 × 10^−4^). ALS5 showed a weak hybridization signal with the hexanucleotide probe that was not visible with the flanking single-copy probe. Conversely the hybridization signal for ALS14 was weaker with the hexanucleotide probe than the flanking probe. In one case (ALS7) the flanking probe detected a smaller expansion product of approximately 3000–4200 bp (130–330 repeats) whereas, the hexanucleotide probe detected a weak signal at the low-resolution area of the agarose gel. Scatter plots of median repeat size versus age at onset (AAO) for both Southern methods suggested a potential trend of increasing repeat size with later AAO (Supplementary Fig. 2). The correlation was more apparent using the hexanucleotide probe (R^2^ = 0.82, F significance = 1.9 × 10^−3^) however, for the single-copy probe ALS7 and ALS14 deviated from this trend (R^2^ = 0.49, F significance = 0.16).

Southern blot analysis of a Gwent kindred gDNA panel (Renton et al., 2011) using the flanking probe identified an approximate 2500 bp product (60 repeats) present in an lymphoblastoid cell line sample from an affected member (NDO6769) that was absent in blood extracted gDNA (Fig. 2A, black arrowhead) and undetectable with the hexanucleotide probe (Fig. 2B). This is possibly because of the clonal selection of B-lymphocytes during Epstein-Barr virus (EBV) transformation. These Southern blotting studies suggest that a subset of cases may show discordant results between different Southern blot protocols, which may be a consequence of sensitivity of the probe, repeat size detection range of the Southern protocol or source of the DNA.

### 3.2 Analysis of *C9ORF72* transcript and protein levels in hexanucleotide repeat expansion carriers

A number of studies have described a reduction in C9orf72 transcript expression as a consequence of the hexanucleotide expansion using qPCR (Ciura et al., 2013; DeJesus-Hernandez et al., 2011; Gijselinck et al., 2012; Mori et al., 2013b). The repeat expansion may predominantly affect variants containing the alternatively spliced exon 1b as its proximal promoter region is predicted to contain the hexanucleotide repeat sequence (DeJesus-Hernandez et al., 2011). We initially examined C9orf72 transcript levels using a panel of qPCR assays designed to target all variants (C9-ALL), long forms (C9-LONG; NM_0.18325.3, NM_001256054.1), short form (C9-SHORT; NM_145005.5), and exon 1b-containing variant 1 (C9-EX1B; NM_0.18325.3). Consistent with previous findings we identified a trend of reduction in C9orf72 transcript levels in large expansion carriers versus controls in all qPCR assays (Fig. 3B), although the reduction was most apparent in the exon 1b-containing variant. This was then confirmed across several brain regions in an independent set of qPCR experiments using a TaqMan probe detecting the exon 1b-containing variant (Fig. 3C). Overall expression was highest in the cerebellum, although variability was high, possibly reflecting tissue-sampling bias. The degree of expression reduction observed in frontal cortex for exon 1b-containing variants was similar in the two qPCR methods. Expression was also significantly reduced in the cerebellum, and there was a trend toward reduced expression in the hippocampus and spinal cord.

Given the presence of the hexanucleotide expansion in the 5′ end of *C9ORF72* between alternative exons 1a and 1b, we then wanted to investigate whether altered exon usage or aberrant splice products occur in expansion carriers. Semiquantitative RT-PCR with exon-flanking primers was used to investigate the ratio between isoforms, brain regions, and genotypes (Fig. 3D). No obvious differences in the isoform ratios, and no new 5′ splice variants were apparent using this method.

To complement the qPCR data we then determined C9orf72 protein levels in our pathological cohort. Characterization of a panel of commercially available C9orf72 antibodies demonstrated a lack of specificity and suitable reactivity for endogenous C9orf72 (Supplementary Fig. 4). Therefore we proceeded with the generation of custom C9orf72 rabbit polyclonal antibodies for immunoblotting studies in pathological material. Specificity of the purified C9-3721 antisera was confirmed using small interfering RNA-mediated knockdown of endogenous C9orf72 in HEK293T cells (Fig. 4A) and mass spectrometry analysis of endogenous C9orf72 immunoaffinity purified from HEK293T cells and postmortem material (peptide information supplied in Supplementary Table 6). Initial immunoblotting of postmortem material and mass spectrometry data identified antibody cross-reaction with GFAP (Fig. 4B, arrowheads) as noted in a previous study (Satoh et al., 2012). This was confirmed by immunoblotting of lysates from cells transfected with recombinant GFAP (Fig. 4C). GFAP cross-reactivity was removed via pre-absorption of C9-3721 through a recombinant GFAP column (Fig. 4B and C). The immunoblotting studies consistently detected approximate 48 kDa and 50 kDa proteins in both cell lines and postmortem brain tissue (Fig. 4A and D, indicated by arrowheads). Small interfering RNA-mediated knockdown of C9orf72 in cell lines suggested that the 48 kDa protein corresponded to the full-length isoform (long form) and the 50 kDa protein may be an additional cross-reaction product. Western blot of postmortem brain tissue detected approximate 27 and 29 kDa proteins of lower abundance that may represent the short isoform (Supplementary Fig. 5B, white arrowheads), however, this was not observed in cell lines. The 48-kDa C9orf72 isoform was found to be of very low abundance in human dermal fibroblasts lines and lymphoblastoid lines and the 27 kDa form was undetectable (data not shown). Quantitative immunoblotting of the 48-kDa isoform in frontal cortex samples analyzed by qPCR identified a significant reduction in C9orf72 protein in repeat expansion cases, although this was not observed in a similar analysis of cerebellar cortex tissue (Fig. 4E). Regression analysis of normalized protein and expression levels of C9orf72 transcripts encoding the long form (C9ORF72-LONG qPCR) suggested evidence of correlation (Supplementary Fig. 6) with an average C9orf72 reduction of approximately 25% in qPCR and immunoblotting studies in frontal cortex. Comparison of expansion size by Southern blot and protein or transcript levels did not indicate a correlation, however, the small number of samples with Southern blot, qPCR and immunoblotting data restricted such an analysis.

## 4 Discussion

Here we compare the use of both single-copy and hexanucleotide-based Southern blotting probes for the detection of *C9ORF72* repeat expansions. Two samples were shown to have short expansion products that were undetectable using the hexanucleotide probe. This highlights the requirement for Southern blotting techniques that can simultaneously detect both large and small repeat expansions independent of repeat composition such as our method or others recently described (Buchman et al., 2013; van Blitterswijk et al., 2013). Southern blot data of gDNA from cell lines should also be interpreted with caution. The observation of reduced modal expansion sizes in cerebellum could reflect the purer sampling of neurons from the granule cell layer, whereas other brain regions such as frontal cortex may contain a greater admixture of nonneuronal cells that may be still mitotic. Our qPCR studies identified a significant reduction in *C9ORF72* expression in both cerebellum and frontal cortex although the effect on other potential pathological markers such as hexanucleotide repeat-containing RNA foci and dipeptide repeat-containing aggregate pathology was not determined.

Two recent Southern blotting studies have observed a correlation of increasing AAO with larger hexanucleotide repeat size (Beck et al., 2013; van Blitterswijk et al., 2013). Analysis within our small cohort also showed a possible trend of increasing AAO with repeat expansion size. This contrasts with a number of noncoding repeat expansion disorders such as Huntington's disease-like 2, fragile X tremor ataxia syndrome, and myotonic dystrophy type one that show evidence of an inverse correlation between age of onset and repeat expansion length (Hamshere et al., 1999; Margolis et al., 2004; Tassone et al., 2007). Although most neurons are postmitotic, the positive AAO and/or repeat length correlation may result from somatic mosaicism where large repeats may expand with age complicating the interpretation of Southern blotting data with relation to clinical features. Such a mechanism has been proposed for the intronic CCTG repeat expansion in *ZNF9* in myotonic dystrophy type 2 where increasing repeat length correlates with age at time of blood withdrawal (Liquori et al., 2001). As we used DNA that was extracted from macroscopically dissected tissue we cannot identify whether the differences arise in neurons or nonneuronal cell populations.

Using complementary qPCR assays in independent experiments, we show evidence of reduced C9orf72 transcript in several brain regions, despite relatively high variability within groups. This may reflect the inherent difficulty of macroscopically dissecting anatomically identical frozen samples for RNA analysis. The reduction of *C9ORF72* expression was most pronounced for the exon 1b-containing variant. This may reflect a specific effect of the hexanucleotide repeat expansion on the promotor region of this variant. Little is known about the significance of the two exon 1a-containing variants, but our semi-quantitative RT-PCR data suggest they are relatively less abundant than isoform 1. Within the sensitivity of our assay, no change in the overall splicing pattern between isoforms was apparent in the presence of the expansion.

One of the key aspects of our study was to quantify C9orf72 transcript and protein levels in postmortem pathological material. To achieve this, we generated and validated a custom C9orf72 antibody and applied it in quantitative immunoblots. We observed a reduction in C9orf72 protein levels in frontal cortex but not cerebellar cortex tissue of cases. However, a significant reduction in exon 1b-containing variants was detected in both tissues by qPCR. Reduced expression of *C9ORF72* has been identified in postmortem brain tissue, lymphoblastoid cell lines, and neurons differentiated from induced pluripotent stem cell (iPSC) lines (Almeida et al., 2013; Ciura et al., 2013; DeJesus-Hernandez et al., 2011; Gijselinck et al., 2012; Mori et al., 2013b). This could be mediated primarily by hypermethylation of a proximal CpG island (Xi et al., 2013) and interaction of the expanded hexanucleotide repeat with trimethylated histones (Belzil et al., 2013). A study using antisense morpholino-mediated knockdown of the Zebrafish *C9ORF72* ortholog resulted in behavioral and cellular deficits including reduced motility and reduced axon length (Ciura et al., 2013). However, recent studies using antisense oligonucleotide mediated depletion of C9orf72 transcripts in iPSC derived neurons and the mouse central nervous system did not observe any adverse effects (Donnelly et al., 2013; Lagier-Tourenne et al., 2013; Sareen et al., 2013). Furthermore, the description of a case homozygous for the *C9ORF72* hexanucleotide repeat expansion that falls within the usual disease spectrum argues against a loss-of-function mechanism (Fratta et al., 2013). The identification of reduced *C9ORF72* expression in ALS and FTLD cases with normal repeat ranges (Ciura et al., 2013) implies that a reduction in C9orf72 levels may also be a common pathogenic factor in ALS or may be a consequence of the disease process.

Although its function is currently unknown bioinformatics studies have identified C9orf72 as a putative member of the DENN domain containing protein family that are known to function as guanine nucleotide exchange factors for Rab GTPases (Levine et al., 2013; Zhang et al., 2012). Existing protein interaction data indicate that C9orf72 may be a component of protein complexes involved in autophagosome initiation (Behrends et al., 2010). Furthermore, a recent study found that neurons differentiated from iPSC cells of *C9ORF72* expansion carriers showed increased sensitivity to chemical inhibitors of endosomal-lysosomal and autophagy pathways compared with controls (Almeida et al., 2013). Dysfunction of vesicular trafficking processes such as endosomal-lysosomal trafficking and autophagy may be a pathological consequence of the *C9ORF72* hexanucleotide repeat and has been implicated in other genetic forms of motor neuron degeneration and FTD (Devon et al., 2006; Filimonenko et al., 2007; Ju et al., 2009; Tumbarello et al., 2013; Wild et al., 2011). A collection of studies analyzing neurons differentiated from iPSCs derived from pathological expansion carriers point toward a gain-of-function mechanism resulting from toxic repeat-containing RNA inclusions and insoluble peptide aggregates generated by non-AUG mediated translation of the hexanucleotide repeat (Almeida et al., 2013; Ash et al., 2013; DeJesus-Hernandez et al., 2011; Donnelly et al., 2013; Lagier-Tourenne et al., 2013; Mori et al., 2013a, 2013b; Sareen et al., 2013). These potentially neurotoxic molecules are a result of bidirectional transcription of the expanded repeat (Gendron et al., 2013; Lagier-Tourenne et al., 2013; Mizielinska et al., 2013; Zu et al., 2013). Disentangling the contribution of these pathological mechanisms awaits the generation of a range of animal and cellular models. The production of a number of sensitive and specific C9orf72 antibodies will also aid in the investigation of such disease models.

## Disclosure statement

Huw R Morris is a co-applicant on a patent application related to C9ORF72-method for diagnosing a neurodegenerative disease (PCT/GB2012/052140).

## Figures and Tables

**Supplementary Fig. 1:**
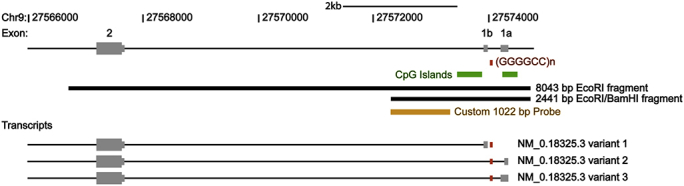


**Supplementary Fig. 2:**
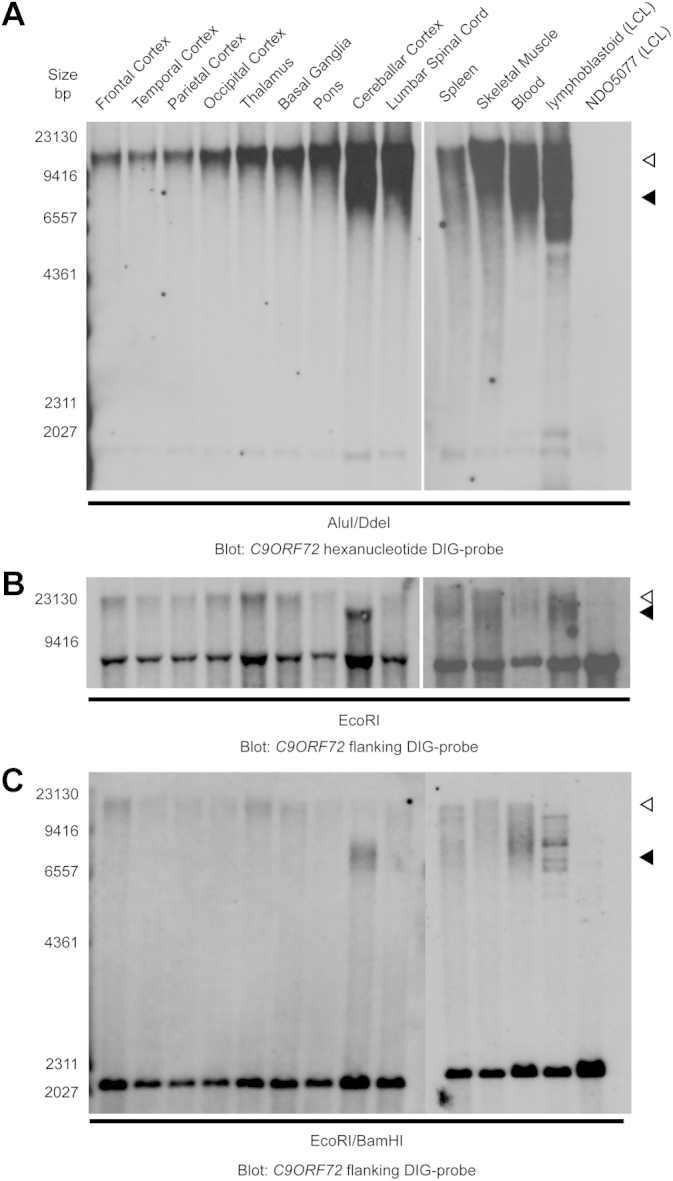


**Supplementary Fig. 3:**
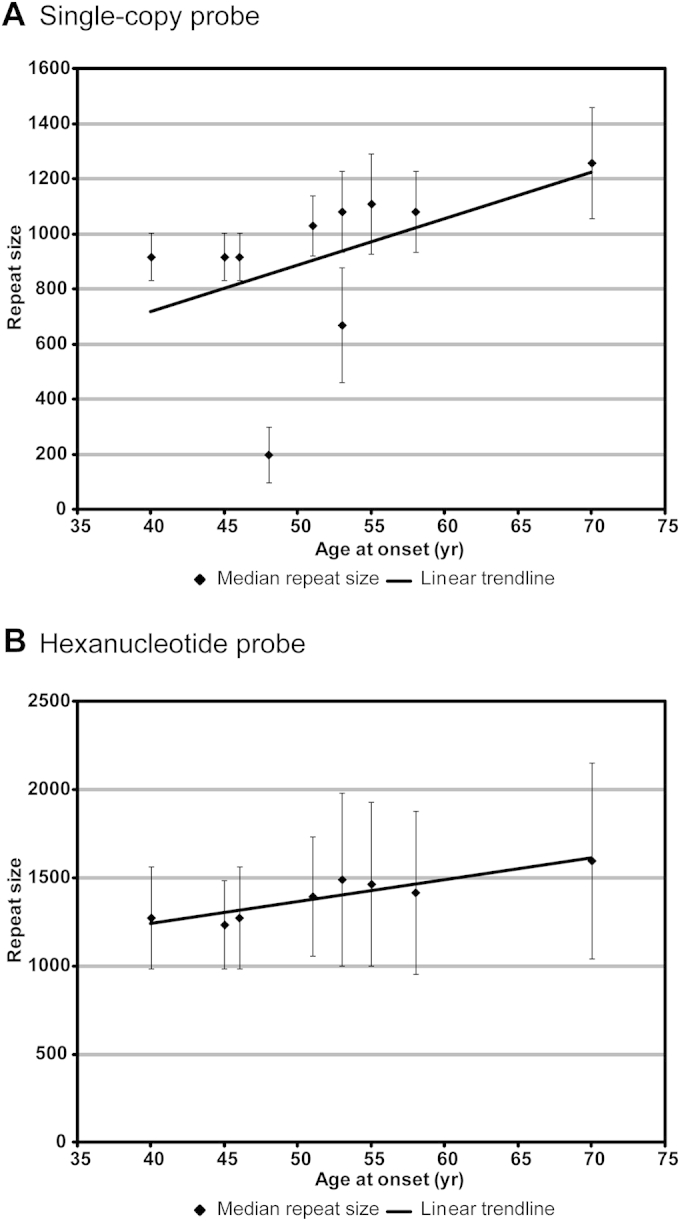


**Supplementary Fig. 4:**
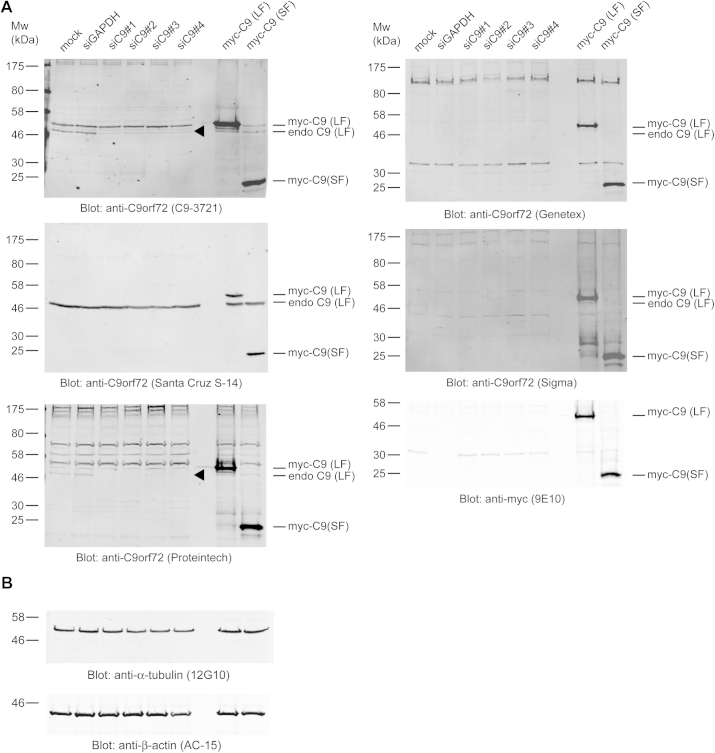


**Supplementary Fig. 5:**
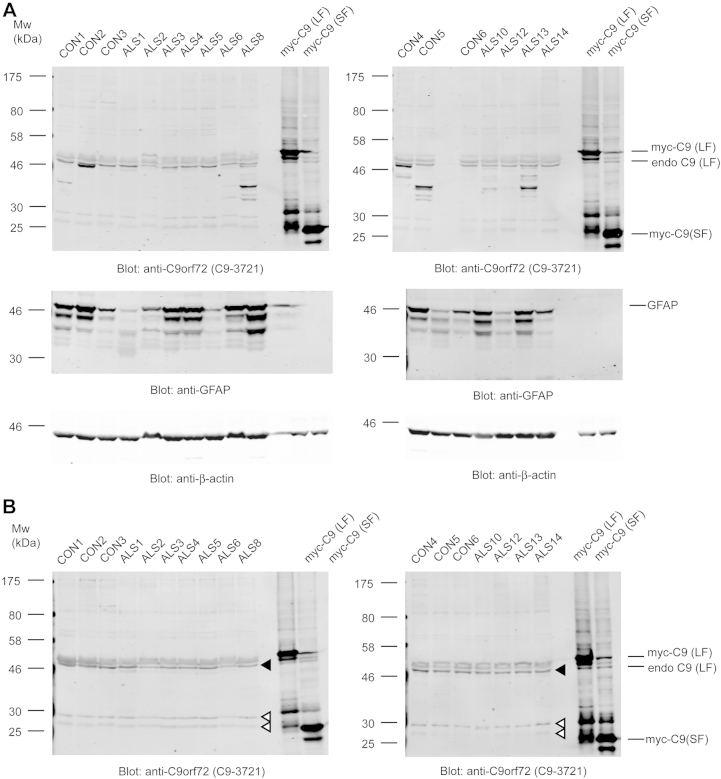


**Supplementary Fig. 6:**
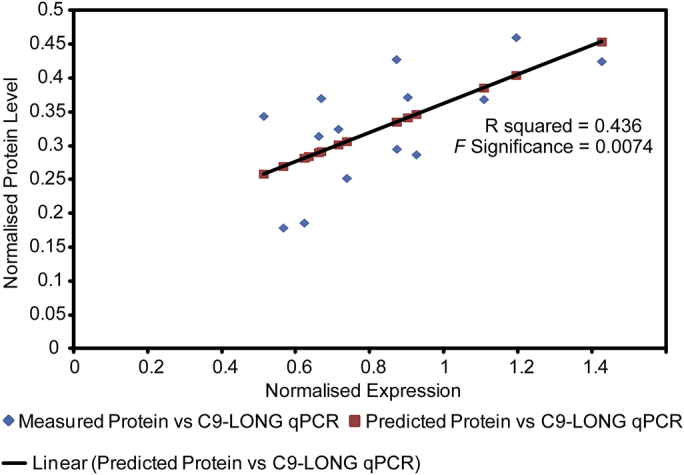


**Fig. 1:**
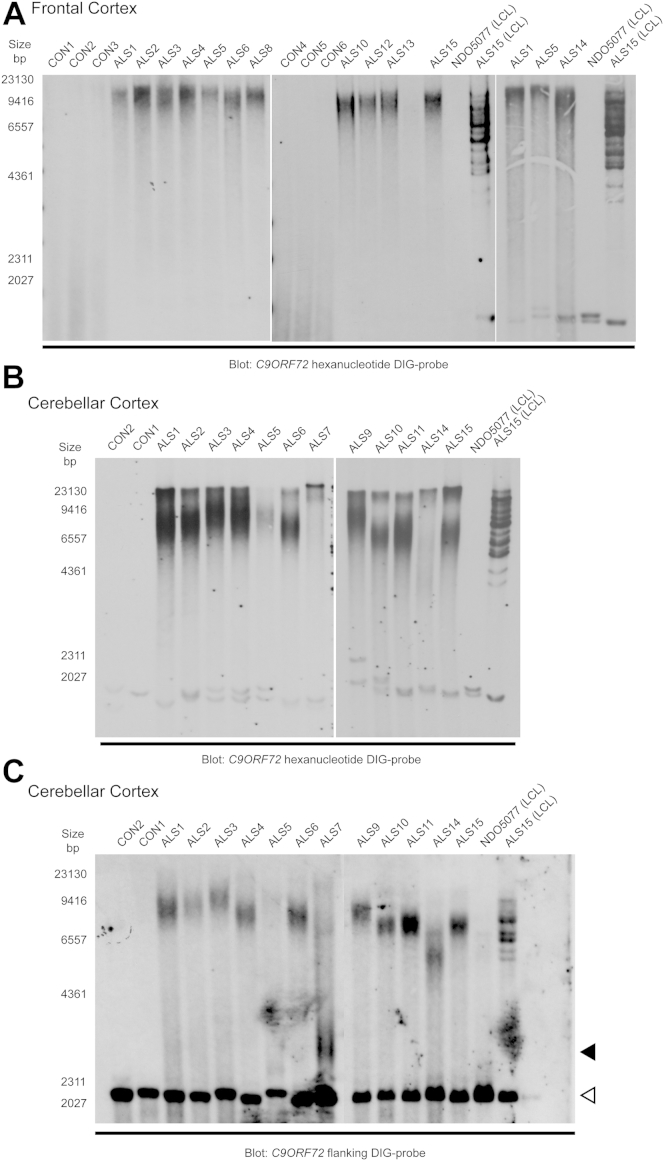
*C9ORF72* repeat expansion analysis of frontal cortex and cerebellar postmortem tissue using 2 Southern blot protocols. Genomic DNA extracted from frontal cortex (A) and cerebellar cortex (B) were digested with AluI/DdeI and analyzed using the published hexanucleotide Southern blot probe. Cerebellar cortex gDNA were also analyzed with a novel flanking Southern blot probe following EcoRI/BamHI restriction digest (C). The flanking probe allows detection of normal range alleles (white arrowhead) that matched the genotypes determined by flanking PCR (Supplementary Table 1). Analysis of path cases (ALS1-15) with the hexanucleotide probe demonstrated the presence of large repeat expansion products (>4000 bp) compared with controls (CON1-6). The expansion products were weaker for ALS5, ALS7, and ALS14. NDO5077 (unaffected control) and ALS15 LCL (expansion positive case) samples were used as internal controls for Southern blots. Analysis of cerebellar tissue with the flanking probe indicated that ALS7 had a reduced modal repeat size of 3000–4200 bp (indicated by black arrowhead). ALS5 had a weaker expansion signal compared with other samples. Abbreviations: PCR, polymerase chain reaction; LCL, lymphoblastoid cell line.

**Fig. 2:**
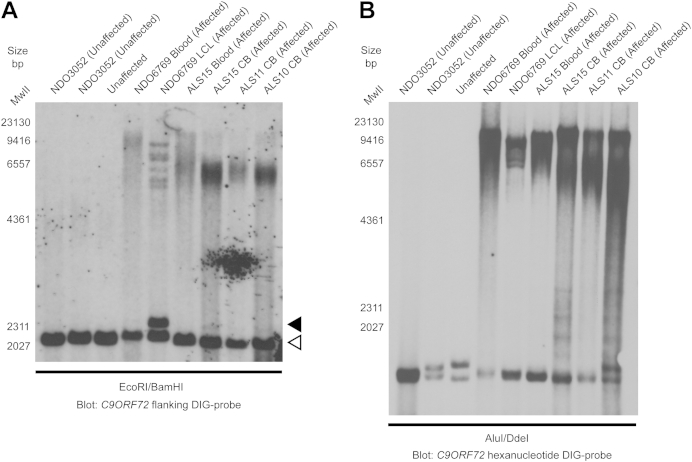
Detection of an LCL specific product in the Gwent kindred affected case NDO6769 (A). Southern blot of Gwent kindred DNA panel using the DIG-labeled *C9ORF72* flanking probe identifies an approximate 60 repeat product in NDO6769 LCL gDNA (indicated with a black arrowhead with normal range alleles marked with a white arrowhead) but not blood extracted gDNA. This product was undetectable with the hexanucleotide probe (B). Abbreviations: CB, cerebellum; DIG, digoxigenin; gDNA, genomic DNA; LCL, lymphoblastoid cell line.

**Fig. 3:**
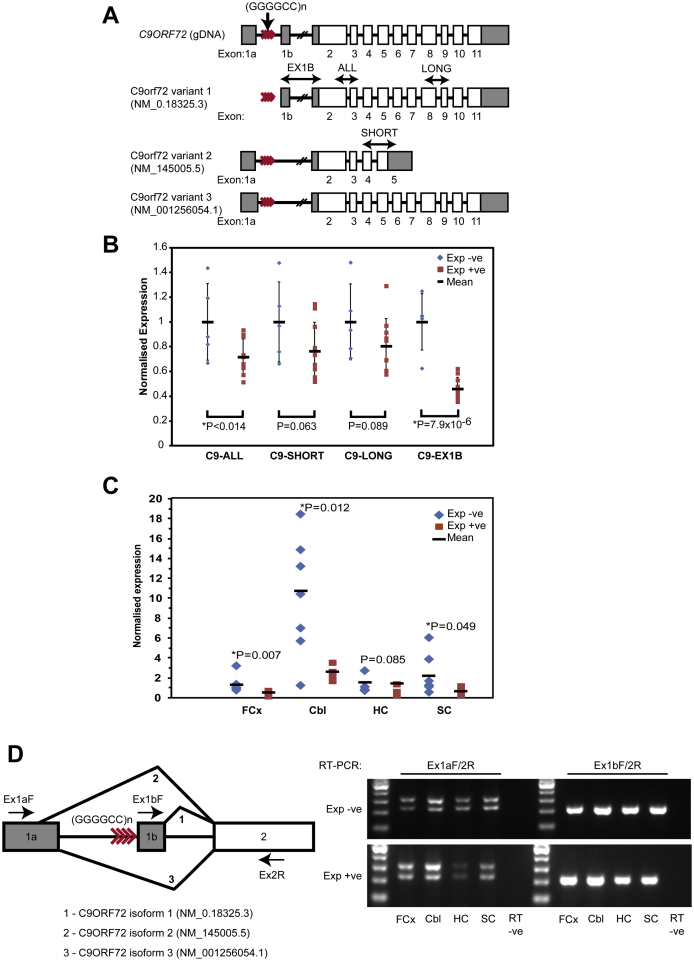
Quantification of *C9ORF72* transcripts in different brain regions. (A) Reported genomic architecture of *C9ORF72* with the location of SYBR green qPCR assays. (B) qPCR analysis of frontal cortex samples (10 cases and 5 controls). Data points show linearized and scaled ΔCt values, error bars show standard deviation with mean expression values as horizontal bars. Statistical significance was determined using 1-tailed *t* tests assuming unequal variance. Cases show a trend of reduced expression across all samples and assays although this is most apparent with exon 1b-containing variant (NM_018325.3). (C) qPCR for exon 1b containing variant 1 (NM_018325.3) shows significantly reduced expression in frontal cortex and cerebellum of expansion carriers (n = 5) compared to expansion negative (n = 8) control cases, as well as a trend toward reduced expression in hippocampus and spinal cord. (D) RT-PCR with forward primers in exon 1a and 1b, respectively, and reverse primer in exon 2 for the detection of variants NM_001256054.1, NM_145005.5, and NM_018325.3 in all examined regions. While NM_018325.3 appears to be more abundant overall, no qualitative difference in the splice variant ratio is apparent between genotypes. Abbreviations: RT-PCR, reverse transcriptase polymerase chain reaction; qPCR, quantitative polymerase chain reaction.

**Fig. 4:**
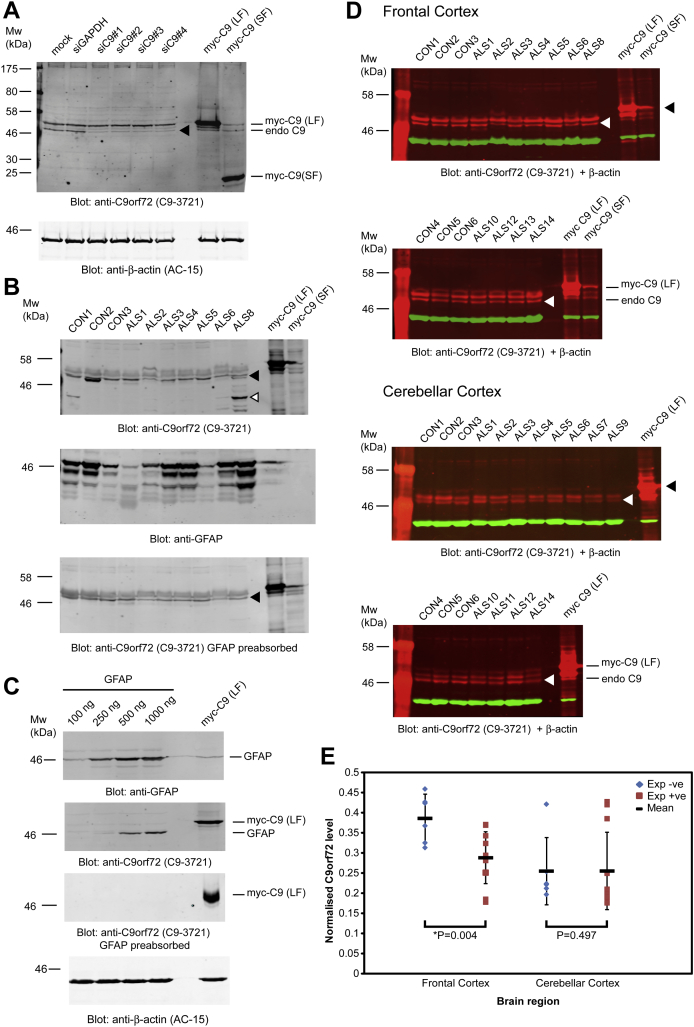
Analysis of C9orf72 protein levels in postmortem tissue using the custom rabbit polyclonal antibody C9-3721. (A) Immunoblotting of HEK293T lysates detects approximate 48 kDa and 50 kDa endogenous protein species. Knockdown of C9orf72 using a panel of siRNA duplexes (siC91-4) leads reduction of the 48-kDa species (black arrowhead, endo C9). β-actin was used a loading control. (B) Initial immunoblots of postmortem frontal cortex tissue detected variable lower molecular weight (white arrowhead) and approximate 48-kDa protein species (black arrowhead) that obscured the endogenous C9orf72 bands. These suspected GFAP-related products were removed using the pre-absorption method. (C) Immunoblot analysis of HEK293Ts transfected with increasing amounts of a GFAP cDNA construct. C9-3721 showed evidence of cross-reactivity with recombinant GFAP that is removed following pre-absorption using a recombinant GFAP column. (D) Example immunoblots of protein extractions from frontal cortex (11 cases and 6 controls) and cerebellar cortex (12 cases and 6 controls) with recombinant myc-tagged C9orf72 long form blot control (myc-C9 LF, black arrowheads) used for LI-COR quantification. Endogenous C9orf72 long form migrates at approximately 48 kDa (endo C9, white arrowheads). (E) Quantification of C9orf72 protein levels using β-actin as a normalizer for total protein. Cases show a significant reduction in the long form compared with controls in frontal cortex using a 1-tailed *t* test assuming unequal variance. This was not observed in cerebellar cortex samples. Abbreviations: cDNA, complementary DNA; GFAP, glial fibrillary acidic protein; siRNA, small interfering RNA.
