# Supplementary material for: Reduced C9orf72 protein levels in frontal cortex of amyotrophic lateral sclerosis and frontotemporal degeneration brain with the C9ORF72 hexanucleotide repeat expansion
Source: Neurobiol Aging. 2014 Jul;35(7):1779.e5–1779.e13. doi: 10.1016/j.neurobiolaging.2014.01.016 (PMC3988882; doi:10.1016/j.neurobiolaging.2014.01.016)
Supplement: Supplementary Data [file mmc1.docx]

**Supplementary Information**

**Supplementary Materials and Methods**

**PCR-based genotyping**

Repeat-primed PCR was performed as previously described (Renton et al. 2011) using alternative primers (Supplementary table 2). Fragment analysis was performed on an ABI 3130xl genetic analyser (Applied Biosystems) using a GeneScan 500 LIZ size standard. Data were analysed using GeneMapper software (version 4, ABI). Repeat expansions produced a characteristic sawtooth pattern. The presence of an expanded *C9ORF72* hexanucleotide repeat was assigned to samples with greater than 30 RP-PCR peaks. Flanking PCR genotyping was performing using the same touchdown PCR procedure as repeat-primed PCR using alternate primers (Supplementary table 2). Repeat numbers were assigned by comparison to samples that were also Sanger sequenced.

**Expression constructs**

Primers for cloning are described in supplementary table 4. Human C9orf72 short and long isoforms were amplified from SH-SY5Y cDNA by PCR. The PCR product was digested and cloned into the *EcoRI*/*NotI* sites of pCMV-myc (Clontech). Constructs were sequencing verified prior to use. The GFAP expression construct was kindly provided by Dr C Tinsley (Cardiff University).

**RNA interference (RNAi)**

27mer RNA oligonucleotide duplexes were designed using predictive algorithms (siDESIGN Center, <http://www.thermoscientificbio.com/design-center/>; siMAX^TM^ Design Tool, <http://www.eurofinsdna.com>) and were selected for their predicted efficacy scores and homology to regions of interest. siRNA duplexes are described in supplementary table 5. siRNAs (Eurofins MWG Operon) were stored as a 10μM working stock in siMAX Universal Buffer (30mM HEPES, 100mM KCl, 1mM MgCl_2_; pH 7.3) at -80°C.

**Cell Culture and transfection**

HEK293T cells were cultured in Dulbecco’s modified Eagle medium (DMEM, Invitrogen) supplemented with 10% (v/v) foetal calf serum (FCS, PAA laboratories) and 1% (v/v) penicillin/streptomycin (PAA laboratories). For overexpression of recombinant C9orf72, 24 h after seeding cells were transfected with myc-C9orf72 expression constructs using Fugene 6 (Promega) according to manufacturer’s instructions. Cell lysates were taken 24 h post-transfection.

For RNAi studies, 24 h after seeding cells were transfected with the different RNAi duplexes (siC9#1-4, siGAPDH or mock) using Lipofectamine® RNAiMAX (Life Technologies) at a final concentration of 10nM. 36 h after the first transfection the cells were re-transfected using the same conditions. The following day, RNA and protein were prepared from each experimental condition.

**Sample preparation**

Proteins were isolated from tissue by homogenisation in treatment buffer (75mM Tris-HCl pH 6.8, 3.8% (w/v) SDS, 4M Urea, 20% (v/v) glycerol) using Lysing Matrix D tubes (MP Biomedicals). The homogenates were clarified by centrifugation (4000 rpm, 10 mins, 4^o^C). Total protein concentration was determined using the BCA protein assay (Thermo Fisher Scientific) as per manufacturer’s instructions. Stocks were diluted to 5 mg/ml using treatment buffer with the addition of 5% (v/v) 2-mercaptuethanol and 0.001% (w/v) bromophenol blue. 75-100 μg of tissue extracts were used for western blotting.

**Antibodies**

Commercial antibodies for C9orf72 were purchased from Santa Cruz Biotechnology Inc (S-14 rabbit polyclonal); Sigma-Aldrich (HPA023873 rabbit polyclonal); GeneTex, Irvine (N1N3 rabbit polyclonal); Abgent (AP12928b rabbit polyclonal); Proteintech (22637-1-AP rabbit polyclonal). Antibodies to cytoskeletal components were purchased from Sigma-Aldrich (anti-β-actin mouse monoclonal AC-15) and Abcam (anti-GFAP ab53554 goat polyclonal). The anti c-myc mouse monoclonal antibody 9E10 was purified in house as described elsewhere (Esapa et al. 2007).

**Immunoaffinity purification and mass spectrometry**

HEK293T cells or post-mortem frontal cortex were solubilised by homogenisation in lysis buffer (50mM Tris-HCl pH 8.0, 1.0% (w/v) SDS, 150mM NaCl, 0.5% sodium deoxycholate, Roche complete EDTA-free protease inhibitor cocktail, 1mM sodium orthovanadate) using a Polytron PT 3100 bench-top homogeniser (Kinematica Inc). The homogenates were diluted with lysis buffer without SDS to a final concentration of 0.25% (w/v) SDS and then clarified by centrifugation (28,000 rpm, 30 mins, 4^o^C). Clarified lysates were pre-cleared with Protein A Agarose (3 h at 4^o^C) prior to immunoaffinity purification using custom anti-C9orf72 rabbit polyclonal antibody conjugated Protein A Agarose beads (overnight at 4^o^C). Immunoaffinity complexes were washed four times with lysis buffer without SDS prior to elution in 1 x LDS sample buffer (Life Technologies). Elutions were supplemented with 50mM DTT final concentration before separation on a 4-12% Bis-Tris Novex gradient gel (Life Technologies). Gel plugs were excised covering the 45-55 kDa range and analysed using the LTQ-Orbitrap mass spectrometry facility (Advanced mass spectrometry facility, Birmingham University, UK). The advanced mass spectrometry facility is supported through the Birmingham Science City Translational Medicine: Experimental Medicine Network of Excellence project*,* aided by Advantage West Midlands (AWM)

**Supplementary figure legends**

**Supplementary figure 1**

Schematic representation of restriction fragment size and position with respect to the custom 1kb southern blot probe and location of the *C9ORF72* hexanucleotide repeat (red box). The probe sequence is located downstream of the hexanucleotide repeat and at the 5’ end of the smaller EcoRI/BamHI restriction fragment. The EcoRI/BamHI digest allows resolution of normal range alleles and potentially small/intermediate range expansions. The EcoRI digest contracts the hexanucleotide expansion ranges allowing increased sensitivity for detection.

**Supplementary figure 2**

Comparative southern blot analysis of *C9ORF72* hexanucleotide expansion size in multiple brain regions and tissues from ALS15 using the hexanucleotide probe (A) and single-copy probe following EcoRI (B) or EcoRI/BamHI (C) digests. The cerebellar tissue tissue shows a reduced modal repeat size range (black arrowhead) compared to other brain regions such as frontal cortex (white arrowhead).

**Supplementary figure 3**

Scatter plot of estimated median hexanucleotide repeat size versus age at onset for cerebellar cortex samples analysed using the single-copy flanking probe (A) and hexanucleotide probe (B). For repeat size estimation the flanking sequence was subtracted from measured restriction fragment sizes (predicted 199 bp for the hexanucleotide probe and the measured reference allele size for the flanking probe). Vertical error bars represent the maximum and minimum range of the expansion signals. Both the single-copy and hexanucleotide probe show evidence of a trend towards increased repeat size with increasing age at onset, although for the flanking probe ALS7 and ALS14 deviate from this trend. Although the pattern of repeat sizes across samples is comparable there is variation in the actual estimated repeat sizes between the two methods.

**Supplementary figure 4**

Characterisation of anti-C9orf72 polyclonal antibodies using siRNA-mediated knockdown. HEK293T cells were transfected with a panel of C9orf72 siRNA duplexes in addition to GAPDH siRNA and mock transfection controls. After 72 h cell lysates were prepared and analysed by western blot using commercial and custom C9orf72 antibodies. (A) C9orf72 western blots. The custom anti-C9orf72 antibody C9-3721 detected a band at approximately 48kDa (black arrowhead, endo C9 LF) in mock transfected and GAPDH siRNA samples that significantly reduced following C9orf72 siRNA treatment (siC9#1-4). The Santa Cruz antibody (S-14) detects major cross-reactive protein at 47-48kDa in HEK293T cells. Only the Proteintech rabbit polyclonal antibody showed evidence of detection of endogenous C9orf72 (black arrowhead, endo C9 LF) with the presence of numerous cross-reactive proteins. All antibodies were capable of detecting recombinant myc-tagged C9orf72 long form (LF) and short form (SF). (B) Western blot detection of α-tubulin and β-actin as loading controls.

**Supplementary figure 5**

Western blot detection of C9orf72 in post-mortem frontal cortex samples using the C9-3721 anti-C9orf72 antibody before and after pre-absorption against GFAP cross-reactivity. (A) Western blot detection of C9orf72 in post-mortem frontal cortex samples using the C9-3721 anti-C9orf72 antibody before pre-absorption against GFAP cross-reactivity. Western blot detection of GFAP (a C9-3721 cross-reactive protein) and β-actin (loading control) was also performed. Additional protein products are detected in the samples that represent full-length and processed forms of GFAP. Surprisingly, the levels of GFAP protein did not seem to correlate with C9-3721 cross-reactivity. (B) Western blots of post-mortem frontal cortex samples with C9-3721 (GFAP pre-absorbed). Endogenous C9orf72 long form (black arrowhead, endo C9 LF) migrates at approximately 48 kDa. The antibody also weakly detected proteins of approximately 27 and 29 kDa (white arrowheads) that may represent processed forms of C9orf72 long form or C9orf72 short form.

**Supplementary figure 6**

Evidence of linear correlation of *C9ORF72*-LONG QPCR and western blot quantified C9orf72 long form using C9-3721 in frontal cortex tissue. Linear regression analysis using the “least squares” method of mean C9ORF72-LONG normalised expression compared to mean normalised Licor intensity for path cohort samples (n = 16). The data shows evidence of a linear trend with *F* statistic significance value 0.0074.

**Supplementary table 1 – Genotyping and basic clinical information for the pathological cohort**

| **STUDY ID** | **RP-PCR (peaks)** | **Flanking PCR Alleles (repeats)** | **ALS** | **FTLD Subtype** | **Age at Onset (yr)** | **FCTX tissue** | **CRBL tissue** | **Blood** | **LCL** |
| --- | --- | --- | --- | --- | --- | --- | --- | --- | --- |
| CON1 | NR | 5 / 8 | Unaffected | Unaffected | n/a | SB, WB, QPCR | SB |  |  |
| CON2 | NR | 2 | Unaffected | Unaffected | n/a | SB, WB, QPCR | SB |  |  |
| CON3 | NR | 2 / 8 | Unaffected | Unaffected | n/a | SB, WB, QPCR |  |  |  |
| CON4 | NR | 2 / 6 | Unaffected | Unaffected | n/a | SB, WB |  |  |  |
| CON5 | NR | 2 / 5 | Unaffected | Unaffected | n/a | SB, WB, QPCR |  |  |  |
| CON6 | NR | 2 / 5 | Unaffected | Unaffected | n/a | SB, WB, QPCR |  |  |  |
| ALS1 | >50 | 2 | FALS | bvFTD | nk | SB, WB | SB |  |  |
| ALS2 | >30 | 2 | FALS | FTD (semantic dementia) | 55 | SB, WB, QPCR | SB |  |  |
| ALS3 | >50 | 10 | SALS | Unaffected | 70 | SB, WB, qPCR | SB |  |  |
| ALS4 | >50 | 8 | SALS | Unaffected | 53 | SB, WB, qPCR | SB |  |  |
| ALS5 | >50 | 15 | SALS | Unaffected | 39 | SB, WB, qPCR | SB |  |  |
| ALS6 | >50 | 2 | SALS | Unaffected | 58 | SB, WB, qPCR | SB |  |  |
| ALS7 | >30 | 2 | FALS | bvFTD | 48 |  | SB |  |  |
| ALS8 | >30 | 11 | Unaffected | bvFTD | 71 | SB, WB, qPCR |  |  |  |
| ALS9 | >50 | 2 | FALS | bvFTD | 51 |  | SB |  |  |
| ALS10 | >50 | 5 | FALS | bvFTD | 45 | SB, WB, qPCR | SB |  |  |
| ALS11 | >50 | 2 | FALS | bvFTD | 40 | SB,qPCR | SB |  |  |
| ALS12 | >50 | 2 | FALS |  | 60 | SB, WB, qPCR |  |  |  |
| ALS13 | >50 | 8 | FALS | bvFTD | 51 | SB, WB, qPCR |  |  |  |
| ALS14 | >50 | 8 | FALS |  | 53 | SB, WB, qPCR | SB |  |  |
| ALS15 | >50 | 2 | FALS | bvFTD | 46 | SB | SB | SB | SB |
| NDO6769 | >50 | 13 | FALS | bvFTD | 44 |  |  | SB | SB |
| NDO5077 | NR | 2 / 10 | Unaffected | Unaffected |  |  |  |  | SB |

Abbreviations are CRBL, cerebellum; FCTX, frontal cortex; NR, normal range; n/a, not applicable; nk, not known; FALS, familial ALS; SALS, sporadic ALS; bvFTD, behavioural variant FTD; LCL, lymphoblastoid cell line; SB, Southern blot; qPCR, quantitative PCR; WB, western blot.

**Supplementary Table 2 - Primers used in genotyping assays**

| **Primer** | **Sequence (5’-3’)** | **Comment** |
| --- | --- | --- |
| ***C9ORF72*-RPPCR-FAM-F** | [6-FAM]GCCTCCTCACTCACCCACT | For repeat-primed PCR |
| ***C9ORF72*-RPPCR-R** | TACGCATCCCAGTTTGAGACGGGGGCCGGGGCCGGGGCCGGGG |  |
| ***C9ORF72*-RPPCR-ANCHOR** | TACGCATCCCAGTTTGAGACG |  |
| ***C9ORF72*-FLANK-FAM-F** | [6FAM]CGCAGCCTGTAGCAAGCTCTGG | For flanking PCR |
| ***C9ORF72*-FLANK-R** | AAACAGCCACCCGCCAGGATG |  |

**Supplementary Table 3 - Primers used in *C9ORF72* RT-PCR and quantitative real-time PCR assays**

| **Assay** | **Primer** | **Sequence (5’-3’)** | **Product (bp)** | **Standard Curve** | | **PCR efficiency (%)** | **ΔC_t_ (target-reference) line slope^1^** |
| --- | --- | --- | --- | --- | --- | --- | --- |
|  |  |  |  | **Slope** | **r^2^** |  |  |
| ***C9ORF72* ALL** | exon 2F | CCCACTTCATAGAGTGTGTGTTG | 124 | -3.196 | 0.994 | 105.6 | 0.047 |
|  | exon 3R | TTCCATTCTCTCTGTGCCTTC |  |  |  |  |  |
| ***C9ORF72* SHORT** | exon 4F | GAAATCACACAGTGTTCCTGAAGAA | 136 | -3.546 | 0.9986 | 92.7 | 0.047 |
|  | exon 5-UTR-R | ATCTGCTTCATCCAGCTTTTATGA |  |  |  |  |  |
| ***C9ORF72* LONG** | exon 8F | CATGGCTCAGGATACGATCA | 101 | -3.271 | 0.995 | 102.2 | 0.050 |
|  | exon 9R | GGAAGGCTTTCACTAGAGTGTCTC |  |  |  |  |  |
| ***C9ORF72* EX1A** | exon 1AF | GGGTCTAGCAAGAGCAGGTG | 82 | -3.372 | 0.992 | 97.9 | 0.029 |
|  | exon 2R | CGACATCACTGCATTCCAAC |  |  |  |  |  |
| **ACTNB** | ACTNB-F | ACGGCCAGGTCATCACCATTG | 92 | -3.312 | 0.998 | 100.4 | n/a |
|  | ACTNB-R | GGAGTTGAAGGTAGTTTCGTGGA |  |  |  |  |  |
| **C9ORF72 EX 1B (NM_018325.3)** | Exon 1B F | CGGTGGCGAGTGGATATCTC | 84 | -3.39 | 0.958 | 97.2 | 0.041 |
|  | Exon 1B R | TGGGCAAAGAGTCGACATCA |  |  |  |  |  |
|  | Exon 1B probe | TAATGTGACAGTTGGAATGC |  |  |  |  |  |
| **C9ORF72 exon 1a** | C9ORF72 exon 1a F | GTCAAACAGCGACAAGTTCCG | 320/242 | n/a |  |  |  |
|  | C9ORF72 exon 2 R | ACCTGTTCTGTCTTTGGAGCC |  |  |  |  |  |
| **C9ORF72 exon 1b** | C9ORF72 exon 1b F | AGGCGCAGGCGGTGGCGAGTG | 221 | n/a |  |  |  |
|  | C9ORF72 exon 2 R | ACCTGTTCTGTCTTTGGAGCC |  |  |  |  |  |

^1^ Validation cut-off for use in ΔC_t_ analysis <0.1

**Supplementary Table 4 – Primers used for cloning**

| **Primer** | **Sequence (5’-3’)** | **Restriction sites** | **Vector** | **Comment** |
| --- | --- | --- | --- | --- |
| **C9orf72-EcoRI-F** | CCC**GAATTC**GGATGTCGACTCTTTGCCCACC | NotI | pCMV-myc | Cloning of C9orf72 (forward primer) |
| **C9orf72-Long-NotI-R** | GAA**GCGGCCGC**ATCATGATTGTGATGGAATAGG | NotI | pCMV-myc | Cloning of C9orf72 long form |
| **C9orf72-Short-NotI-R** | GAA**GCGGCCGC**ATCTGCTTCATCCAGCTTTTATGA | NotI | pCMV-myc/pET32b | Cloning of C9orf72 short form |
| **C9orf72-PET-F** | CCC**GAATTC**AATGCCATCAGCTCACACTTG | EcoRI | pET32b | Cloning of C9orf72 short form in pET32b |
| **GFAP-EcoRI-F** | CCC**GAATTC**ATGGAGAGGAGACGCATCAC | EcoRI/SalI | pET32a | GFAP fusion for antibody pre-absorbance |
| **GFAP-SalI-R** | CCC**GTCGAC**TCACATCACATCCTTGTGCTC |  |  |  |
| ***C9ORF72*-SB-Probe-F** | AACCGTCCACTTTCCACAAC | n/a | pGEM-T | Cloning of *C9ORF72* flanking probe for southern blot |
| ***C9ORF72*-SB-Probe-R** | TCACAGCTCCGAGATGACAC | n/a |  |  |

**Supplementary Table 5 – siRNA duplexes**

| **Primer** | **Sequence (5’-3’)** | **C9orf72 target exon(s)** | **Isoforms targeted** |
| --- | --- | --- | --- |
| **siC9#1** | GAAAGGAAGAAUAUGGAUGCAUAAGGA | Exon 2 and 3 | Pan isoform |
| **siC9#2** | GGAAGAAUAUGGAUGCAUAAGGAAAGA | Exon 2 and 3 | Pan isoform |
| **siC9#3** | UACAGUACUCAAUGAUGAUGAUAUUGG | Exon 5 | Pan isoform |
| **siC9#4** | AGAAGAAGACAUGGCUCAGGAUACGAU | Exon 8 | Long form specific |
| **siGAPDH** | CGGAGUCAACGGAUUUGGUCGUAUUGG |  |  |

**Supplementary table 6**

**A - Post-mortem frontal cortex**

| **Identification** | **NCBI Ref** | **GI Accession** | **Unique peptides** | **Score** | **Coverage (%)** | **No. peptides** | **Mw (kDa)** |
| --- | --- | --- | --- | --- | --- | --- | --- |
| Glial fibrillary acidic protein isoform 1 [Homo sapiens] | NP_002046.1 | 4503979 | LDQLTANSAR  LEAEnNLAAYR  EAASYQEALAR  EAASYqEALAR  DNLAQDLATVR  ALAAELNQLR  ITIPVQTFSNLQIR  LADVYQAELR  FADLTDAAAR  LDQLTAnSAR  HLQEYQDLLNVK  LEAENNLAAYR  QLQSLTcDLESLR  ALAAELnQLR  LADVYqAELR  LEVERDNLAQDLATVR  ALAAELnqLR  FASYIEK  LEEEGQSLKDEmAR  LQDETNLR  LDqLTAnSAR  ELQEQLAR  LEAEnnLAAYRQEADEATLAR  LEAENNLAAYRqEADEATLAR  IESLEEEIR  ELQEqLAR  LRLDQLTANSAR  DNLAqDLATVR  QKLQDETNLR  mPPPLPTR  QQVHVELDVAKPDLTAALK  LEAENNLAAYRQEADEATLAR  KIHEEEVRELQEQLAR  QEADEATLAR | 551.26 | 52.09 | 23 | 47.3 |
| Uncharacterised protein C9orf72 Isoform a [Homo Sapiens] | NP_060795 | 37039612 | YESGLFVQGLLK  SELTAFWR  TLcLFLTPAER  DSTGSFVLPFR  IILEGTER  AFLDQVFQLKPGLSLR  LcEAESSFK | 44.93 | 15.59 | 7 | 54.3 |

**B – HEK293T**

| **Identification** | **NCBI Ref** | **GI Accession** | **Unique peptides** | **Score** | **Coverage (%)** | **No. peptides** | **Mw (kDa)** |
| --- | --- | --- | --- | --- | --- | --- | --- |
| Uncharacterised protein C9orf72 Isoform a [Homo Sapiens] | NP_060795 | 37039612 | YESGLFVQGLLK  AFLDQVFQLKPGLSLR  TLcLFLTPAER  QVmYAPYPTTHIDVDVNTVK  DSTGSFVLPFR  TEIALSGK  SELTAFWR  IILEGTER  FFVLSEK  LcEAESSFK  KALTLIK  mEDQGQSIIPmLTGEVIPVmELLSSmK | 208.39 | 29.94 | 12 | 54.3 |
